# Circulating Tumor Cell PD-L1 Expression as Biomarker for Therapeutic Efficacy of Immune Checkpoint Inhibition in NSCLC

**DOI:** 10.3390/cells8080809

**Published:** 2019-08-01

**Authors:** Vera Kloten, Rita Lampignano, Thomas Krahn, Thomas Schlange

**Affiliations:** Precision Medicine Markers, Pharmaceutical Division, Bayer AG, 42113 Wuppertal, Germany

**Keywords:** liquid biopsy, CTCs, immune checkpoint inhibitors, PD-L1 expression, NSCLC

## Abstract

Over the last decade, the immune checkpoint blockade targeting the programmed death protein 1 (PD-1)/programmed death ligand 1 (PD-L1) axis has improved progression-free and overall survival of advanced non-small cell lung cancer (NSCLC) patients. PD-L1 tumor expression, along with tumor mutational burden, is currently being explored as a predictive biomarker for responses to immune checkpoint inhibitors (ICIs). However, lung cancer patients may have insufficient tumor tissue samples and the high bleeding risk often prevents additional biopsies and, as a consequence, immunohistological evaluation of PD-L1 expression. In addition, PD-L1 shows a dynamic expression profile and can be influenced by intratumoral heterogeneity as well as the immune cell infiltrate in the tumor and its microenvironment, influencing the response rate to PD-1/PD-L1 axis ICIs. Therefore, to identify subgroups of patients with advanced NSCLC that will most likely benefit from ICI therapies, molecular characterization of PD-L1 expression in circulating tumor cells (CTCs) might be supportive. In this review, we highlight the use of CTCs as a complementary diagnostic tool for PD-L1 expression analysis in advanced NSCLC patients. In addition, we examine technical issues of PD-L1 measurement in tissue as well as in CTCs.

## 1. Immune Checkpoint Blockade Therapy in Non-Small Cell Lung Cancer (NSCLC): State of the Art

Lung cancer is the most common cancer in men and the third frequent cancer in women worldwide. With a poor five-year survival rate of 10–15%, lung cancer is the major cause of cancer-related deaths [1]. Nowadays, molecular testing in advanced non-small cell lung cancer (NSCLC) patients includes screening for targetable alterations, e.g., *EGFR* mutations or *ALK* rearrangements, and, in addition, factors predictive of response to immunotherapy, thus, immune checkpoint inhibitors (ICIs). The introduction of ICIs in the clinic has led to increasing response rates in locally advanced and metastasized NSCLC [2,3,4,5]. ICIs are designed to target inhibitory checkpoint molecules, such as programmed cell death protein 1 (PD-1), and its ligand programmed cell death protein ligand 1 (PD-L1). PD-L1, a type I transmembrane protein with an extracellular N-terminal domain, inhibits the immune response through interaction with its receptor, PD-1, expressed among other immune cells on activated T- and B-cells [6]. Thereby, PD-L1 upregulation in tumor tissue enables evasion of immune surveillance by the inhibition of immune cell activation. In contrast to conventional therapies that directly target cancer cells, anti-PD-1/PD-L1 antibodies reactivate the immune system of patients to eradicate tumors, which induces durable and long-lasting antitumor immunity in patients with different tumor types, including lung cancer [7].

**Figure 1:**
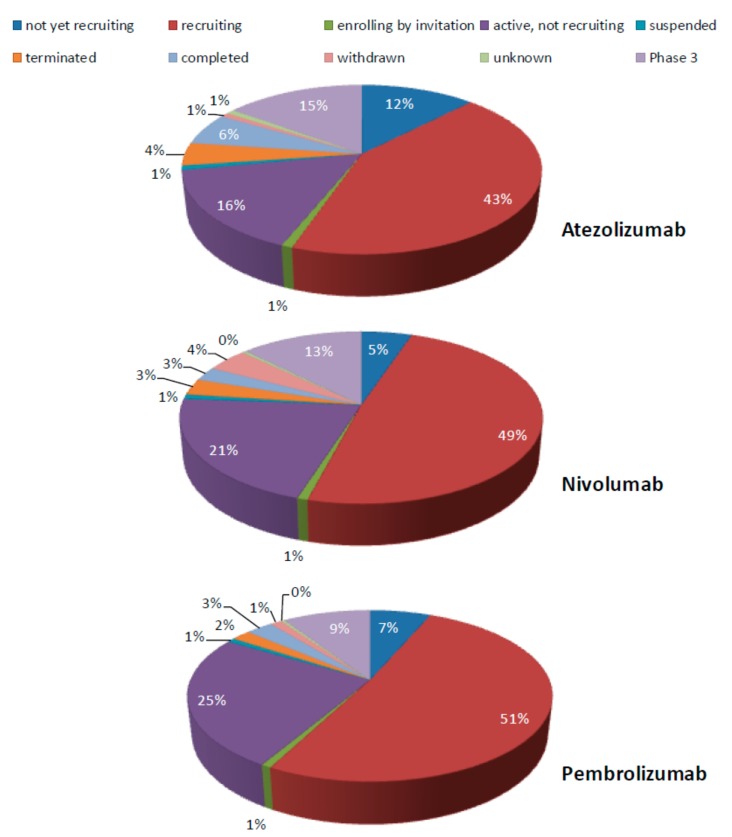
Overview of listed clinical trials for Atezolizumab, Nivolumab, and Pembrolizumab in lung cancer (data based on ClinicalTrial.gov).

As a biomarker for selection of patients eligible for ICI therapy, the PD-L1/PD-1 axis has been investigated in many studies [8]. Today, two antibodies blocking PD-1, Nivolumab (Opdivo, Bristol-Myers Squibb) and Pembrolizumab (Keytruda, MSD SHARP and DOHME GMBH) as well as one antibody targeting PD-L1, Atezolizumab (Tecentriq, Roche) [9,10], have US Food and Drug Administration (FDA) approval in NSCLC. In more detail, Pembrolizumab has approval for both first- and second-line treatment, while Nivolumab also has third-line approval for NSCLC. In addition, there are ongoing studies for the use of Atezolizumab in the third-line setting as well. This has led to an increased interest in potential additional clinical applications for these therapeutics. Currently, there are 97 listed clinical trials for Atezolizumab, 203 trials for Nivolumab, and 225 trials for Pembrolizumab in lung cancer (status June 3rd 2019, extracted from ClinicalTrial.gov [11] (Figure 1). Search terms and synonyms are listed in Tables S1–S3. Most of the studies are recruiting patients or are under investigation. For Atezolizumab, 15% of the studies are in phase III, while for Nivolumab and Pembrolizumab, 13% and 9%, respectively, are listed as phase III trials. Based on ClinicalTrial.gov, 3% of studies with Nivolumab, 6% of studies with Atezolizumab, and 3% with Pembrolizumab treatment have been completed (Figure 1). The efficacy of immune checkpoint inhibitors alone or in combinations in NSCLC has been summarized in detail elsewhere [12,13]. 

Not all patients with advanced NSCLC benefit from these drugs. Only 20% of unselected NSCLC patients show a response to ICIs (summarized e.g., in [14]), underlining the necessity to select the right patients. The gold standard for treatment decision is an immunohistochemistry (IHC)-based companion or complementary diagnostic tests for PD-L1. Two anti-PD-L1 antibodies, Ventana PD-L1 (SP142) assay (Ventana Medical Systems, Inc, Tucson, AZ, USA and PD-L1 IHC 22C3 pharmDx (Dako North America, Inc. Santa Barbara, CA, USA) have FDA approval for PD-L1 IHC. However, PD-L1 expression assessed by IHC requires a tissue sample which can be insufficiently representative of overall tumor/metastasis expression or not available from patients, thus impeding treatment decision-making. In addition, the PD-L1 expression may be considerably heterogeneous across tumor boundary to core [15]. Furthermore, dynamic changes of PD-L1 expression in tumor cells might occur before or during therapy with PD-1/PD-L1 inhibitors, leading to different sensitivity to PD-1/PD-L1 blockade which would similarly be missed by a single biopsy. With the increasing number of therapeutic regimens and targeted therapies, molecular profiling of NSCLC is becoming crucial at every step of disease progression to reveal the biological alterations that are driving resistance and impact on treatment decisions. Obtaining serial tumor tissue biopsies is highly challenging and often not feasible. Such patients may benefit from a molecular characterization of PD-L1 expression in circulating tumor cells (CTCs) extracted from blood. In the last decade, molecular analysis of CTCs in body fluids (as a “liquid biopsy”) started to have a growing impact on the clinical management of cancer patients. Today, liquid biopsy is a rapidly expanding field in translational cancer research, and it shows the potential to complement diagnostic and therapeutic care of cancer patients [16]. CTCs hold promise to better reflect tumor heterogeneity compared to tissue biopsies because they might originate from different tumor sites and reflect properties from the primary tumor site as well as from metastatic sites shown, e.g., in breast cancer [17]. In addition, they could lead to important insights on how tumor cells become resistant to immune therapy because they can be analyzed longitudinally as liquid biopsies. Interestingly, circulating tumor DNA (ctDNA) is currently under investigation in several clinical trials as a biomarker for tumor mutation burden (TMB) rather than CTCs. There is strong interest in TMB since a positive correlation between TMB in tumor tissue and a clinical benefit from immunotherapy has retrospectively been observed [18]. However, there are several publications implying clinical relevance of PD-L1-positive CTCs in cancer including NSCLC [19,20,21,22].

## 2. Clinical Significance of PD-L1-Positive CTCs in NSCLC

Despite the reported value of assessing the overexpression of PD-L1 on cells of different types in solid tumors—including lung cancer—as a promising marker to predict anti PD-1/PD-L1 treatment efficacy, the predictive value of PD-L1 expression is still controversial and related investigations face the three major limitations of tissue biopsies: Invasiveness, sampling error due to tumor heterogeneity, and mostly unfeasible longitudinal sampling. To overcome these issues and support histological analysis, the expression of PD-L1 has been explored on CTCs and has been correlated to patients’ clinical outcomes (Table 1). The clinical significance of PD-L1-positive (PD-L1^+^) CTCs in NSCLC is to date in its infancy as the first related study was published in 2016 by Schehr and colleagues [23], who initially focused on technical optimizations of PD-L1^+^ CTC enrichment. Schehr et al. reported the presence of a population of co-isolated CD11b^+^ (marker for myeloid development), CD45-low, and cytokeratin-positive (CK^+^) cells—via an in-house produced immunomagnetic enrichment system—misidentified as CTCs and expressing PD-L1. The quantification of PD-L1 could therefore be skewed by false positive events, thus requiring careful analysis in order to increase the accuracy of the assay [23]. However, one has to be aware that inconsistency might be also caused by various types of therapy since patients in this study were treated with mainly radio- and/or chemotherapy before blood collection, followed by a first- to fifth- line of therapy with ICIs. On the same line, Bao and colleagues [24] also focused on the development and optimization of a CTC-sorting system—in this case a size-based chip—which could give the chance to investigate the PD-L1 expression, achieved through a RT-qPCR approach. However, the lack of CTC^+^ patients (~7% based on CK19 mRNA expression) did not allow for drawing any significant conclusion about the clinical utility of PD-L1 [24].

The predictive utility of PD-L1^+^ CTCs in a chemotherapy setting has been further investigated by Kallergi and colleagues [25]. In this study, the ISET (Isolation by SizE of Tumor cells, Rarecells Diagnostics SAS) technology followed by Giemsa and immunostaining was utilized to detect PD-1^+^ and PD-L1^+^ CTCs in metastatic NSCLC patients before (chemotherapy-naïve, *n* = 30) and after chemotherapy (after the third chemotherapy cycle at the time of assessment of treatment efficacy, *n* = 11). Giemsa staining revealed CTCs in 28 of 30 (93.3%) patients at baseline and in 9 of 11 (81.8%) patients studied after the third chemotherapy cycle with a median of 5 CTCs/mL of blood (range, 0–23 CTCs/mL of blood). Of interest, using immunostaining, CTCs could be detected in 17 of 30 (56.7%) patients at baseline and in 8 of 11 (72.7%) after the third treatment cycle. The concordance between the two detection methods at baseline and after the third treatment cycle was 63.3% and 67%, respectively. The rate of detection was 30% (9 of 30) and 27% (8 of 30) before treatment, and in 9% (1 of 11) and 46% (5 of 11) after 3 cycles of chemotherapy, respectively for PD-1^+^ and PD-L1^+^ CTCs. Interestingly, an increase of 20% PD-L1^+^ CTCs (*p* = 0.096) and a decrease of 21% PD-1^+^ CTCs (*p* = 0.785) after chemotherapy was observed. In addition, a shorter progression-free survival (PFS) could be observed for patients with >3 PD-1^+^ CTCs at baseline (*p* = 0.022) but not for PD-L1 expressing tumor cells, thus suggesting a potential clinical role for PD-1^+^ CTCs rather than for PD-L1^+^ CTCs [25].

Through a different size-based CTC-enrichment approach (CellSieve™ Microfiltration Assay, Creatv MicroTech) followed by immunostaining, Adams et al. [26] investigated the expression of PD-L1 in different CTC subtypes, i.e., PDCTCs (prognostically relevant pathologically definable CTCs), EMTCTCs (CTCs undergoing epithelial-to-mesenchymal transition), and CAMLs (cancer-associated macrophage-like cells) in a prospective pilot study with 41 NSCLC patients (stage I–IV) undergoing radiotherapy, while 34% (14 of 41) received prior chemotherapy. The researchers were able to identify at least one CTC (i.e., PDCTC, EMTCTC, or CAML) in 35 of the 41 samples (85%) at baseline and in all 41 samples (100%) at a follow-up sample taken two to three weeks after radiotherapy initiation. Specifically, EMTCTCs were found in 49% of baseline samples and in 66% of follow-up samples. CAMLs were found in 81% of baseline samples and in 100% of follow-up samples. PDCTCs were found in only one sample at baseline (2%) and in only three samples at follow-up (7%). Furthermore, Adams and colleagues confirmed an intra- and interpatient dynamic expression of PD-L1 in CTCs before and after therapy. The researchers reported 51% patients exhibiting no/low PD-L1 expression at baseline and follow-up, 17% had persistently medium/high at the two time points, and 32% patients showing an increase in PD-L1 expression in CTCs from low to medium in the follow-up visit. Furthermore, concordance between CTCs and matched tumor tissue was highly depending on the antibody clone utilized for immunohistochemistry (IHC) and also, given the restricted amount of patients, no statistical analysis could be performed. However, a sequential PD-L1 evaluation in patients two to four months after the end of radiotherapy, exhibited that 87% expression of the marker was unchanged, suggesting the importance of longitudinal analysis of PD-L1 expression in CTCs [26].

The impact of radiation therapy on PD-L1 expression in CTCs was recently investigated by Wang et al. [27] monitoring the dynamic changes of PD-L1 expression in CTCs of 13 nonmetastatic NSCLC patients who received radiation alone (*n* = 5) or chemoradiation (*n* = 8). Serial blood samples from the patients enrolled in the study were collected before the initiation of radiation, during radiation, and at follow up, approximately one month after radiation (*n* = 38 samples). CTCs were detected in all 38 samples with an average of 21.3 CTCs/mL (range of 4–72 CTCs/mL), while PD-L1^+^ CTCs were detected in 24 (66.7%) out of 36 samples analyzed, ranging from 0 to 43 PD-L1^+^ CTCs/mL. In line with the results by Adams et al. [26], patients treated with radiation or with concurrent carboplatin and paclitaxel had increased PD-L1^+^ CTCs during treatment (PD-L1^+^ CTC% was higher in visit two than that in visit one (median 0.7% vs. 24.7%, *p* = 0.0068)). In addition, PD-L1^−^positive patients had a shorter PFS compared to PD-L1-negative patients using a PD-L1^+^ CTC cut-off ≥5%. Notably, one of the patients who had a high PD-L1^+^ CTC count at visit two and visit three was put on therapy with Pembrolizumab after initial progression and has had stable disease for seven months [27], implying that patients who become (re-)sensitized to ICIs can be identified by PD-L1 CTC expression analysis.

The largest studies so far, investigating the role of PD-L1^+^ CTCs in the clinical setting, were conducted by Ilié et al. [28] and Janning et al. [29], who utilized a sized based CTC-enrichment approach (Isolation by SizE of Tumor cells (ISET), Rarecells Diagnostics SAS) with, respectively, the FDA-approved EpCAM-based CellSearch^®^ System (Menarini Silicon Biosystems Inc, Huntingdon Valley, PA, USA and the epitope-independent Parsortix^TM^ system (Angle, Guildford, UK) followed by immunostaining of retained cells. In the study of Ilié and colleagues, CTCs were detected in 80 of 106 (75%) patient samples, while 99% (79 out of 80) CTC-positive samples exhibited more than 5 CTCs per 4 mL blood, with a median of 60 CTCs per 4 mL (range: 2–256 CTCs/4 mL). Furthermore, the researchers extracted ≥1 PD-L1^+^ CTCs in 8% of patients with advanced stage III and IV NSCLC (*n* = 6/71 samples) with 93% concordance to PD-L1^+^ tumor cells of matched primary tissue (specificity = 100%; sensitivity = 55%). In addition, they could observe a trend towards poor clinical outcomes in patients with PD-L1^+^ CTCs receiving first line of chemotherapy, similar to the trend observed for PD-L1^+^ primary tumors [28]. Janning and colleagues detected ≥1 CTC in 68.5% (*n* = 61/89 samples) and ≥3 CTCs in 33.7% (*n* = 30/89 samples) of NSCLC (mostly stage IV) patients using the Parsortix^TM^ system. Thereof, the researchers found ≥1 PD-L1^+^ CTC in 56% (*n* = 50/89 samples) and ≥3 PD-L1^+^ CTCs in 26% (*n* = 23/89 samples) of patients. Amongst patient samples with at least three CTCs (CD45^−^/K^+^), 47% (14/30) harbored exclusively PD-L1^+^ CTCs and 47% (14/23) had both PD-L1^+^ and PD-L1^−^ CTCs [29]. Of interest, the percentage of PD-L1^+^ CTCs did not correlate with the percentage of PD-L1^+^ tumor cells in primary tumor tissue biopsies determined by immunohistochemistry (*p* = 0.179). In patients undergoing therapy with Pembrolizumab, Nivolumab, or Atezolizumab the researchers indicated that in 89% of the responding patients either a decrease or no change of their total CTC counts after three or five cycles of therapy (decrease: 6/9; no change 2/9, increase: 1/9) was shown. In contrast, upon disease progression, all patients showed an increase in PD-L1^+^ CTCs [29]. 

The predictive value of PD-L1^+^ CTCs in NSCLC patients under immunotherapy has also been investigated in several other studies. Nicolazzo and colleagues [30] first focused on the evaluation of PD-L1^+^ CTCs utility in patients with stage IV NSCLC treated with the anti-PD-1 Nivolumab. By utilizing the CellSearch^®^ system as a CTC enrichment approach, they reported a CTC detection rate of more than the 40% usually described by the literature [31], with an extremely high frequency of PD-L1 expression (95%) in 83% of the patients at baseline. The number of CTCs detected ranged from 1 to 20 (median number of CTCs 5.2). After three months of treatment, the fraction of PD-L1^+^ CTCs ranged from 25% to 100%, while after six months 50% showed PD-L1^+^ CTCs. Therefore, even though both the presence of CTCs and the PD-L1 expression were associated with poor clinical outcomes (statistics not available), the lack of patients with PD-L1-negative CTC fractions did not allow for any conclusions about the real prognostic and predictive relevance of this marker [30]. In addition to Nicolazzo et al., Guibert and colleagues [19] detected PD-L1^+^ CTCs in 93% of advanced NSCLC patients before Nivolumab treatment, with a median proportion of CTCs expressing PD-L1 of 17.2% using the ISET technology followed by immunostaining. Interestingly, no correlation could be observed with PD-L1+ tissue biopsies (72%; *r* = 0.04, *p* = 0.77). Furthermore, in a study by Kulasinghe and colleagues [20], 66% of NSCLC patients exhibiting PD-L1^+^ CTCs (64.7%; *n* = 11)—enriched through the size-based ClearCell FX—were treated with Nivolumab, but no correlation between PD-L1 expression and clinical outcomes could be observed. The impact of the epithelial-to-mesenchymal transition (EMT) of CTCs in NSCLC patients under Nivolumab treatment was described in a recent study by Raimondi et al. [32] using the filtration technology ScreenCell. The researchers investigated 13 patients with metastatic NSCLC progressing post-prior systematic treatment with Nivolumab. They found ≥1 CTC 69% (9 of 13) of patients with a percentage of 5% to 80% of PD-L1^+^ CTCs. Interestingly, PD-L1 was found coexpressed with EMT markers in a percentage of cells that was ranging between 50% and 78%. This might provide a biologic explanation for the persistence of PD-L1-positive CTCs in NSCLC patients after six months of treatment, predicting resistance to the anti-PD-1 Nivolumab shown by Nicolazzo and colleagues [30].

Beside the potential predictive role of PD-L1+ CTCs for Nivolumab treatment, its clinical significance has also been investigated for immunotherapy based on Pembrolizumab (PD-1 inhibitor) by Dhar and colleagues [33], who also opted for a size-based CTC enrichment system (Vortex HT chip) followed by immunostaining of captured cells. In this study, ≥1 PD-L1+ CTC were detected in ~97% patients before treatment with a discrete concordance with tissue biopsy. However, due to the restricted number of primary tumor biopsies available (*n* = 4), statistical analysis was not possible. Importantly, patients with >50% PD-L1+ CTCs (*n* = 3/4) experienced an improved progression-free survival under Pembrolizumab treatment, indicating the need for further confirmation of the available data to reconcile the conflicting evidence.

In summary, despite the limited amount of studies published to date, these first results imply PD-L1+ CTCs might play a role in determining response to different ICI therapeutic approaches (summarized in Table 1).

Several studies showed that the efficacy of PD-1/PD-L1 blockade could be also affected by PD-L1 expression on tumor-infiltrating cells in different types of cancer, including lung cancer. Herbst et al. [34] showed across multiple cancer types that responses were observed in patients with tumors expressing high levels of PD-L1, especially when PD-L1 was expressed by tumor-infiltrating immune cells. In another study by Kim et al. [35], it was shown that increased numbers of CD8^+^ or PD-1^+^ tumor-infiltrating lymphocytes (TILs) were significantly associated with prolonged disease-free survival of these patients, whereas PD-L1 and PD-L2 expression had no significant prognostic implications. He and colleagues [36] revealed a 43.2% positive PD-1 staining on TILs in NSCLC tumor tissue, while PD-L1 was detected on both tumor cells and TILs. Studies investigating the relation between PD-1 and PD-L1 in lung cancer were focused on tumor tissue samples facing the same issues as mentioned above. 

However, it has to be noted that even though the field of CTCs carries a great potential with liquid biopsy to better integrate the heterogeneous and potentially dynamic expression of PD-L1 in the course of NSCLC pathology, it still requires a series of (pre-)analytical standardizations, in order to avoid misinterpretations and guarantee a reliable clinical treatment decision [37]. 

## 3. The Need for (Pre-)Analytical Standardizations

Important aspects need to be taken into consideration when comparing these studies. First and foremost, a detailed report about patients´ clinical data (e.g., TNM classification, grade) followed by more technical standardization regarding the sampling, blood stabilization, storage time and temperature, and CTC enrichment and detection approach, as well as the antibody cocktail utilized for the immunostaining and the threshold applied for the PD-L1 positivity are necessary (Table 1). Indeed, variations and lack of consensus in all these steps could lead to major discrepancies among studies, thereby hampering a proper comparison of results and clinical applications in the near future [38]. The advent of anti-PD-L1 antibodies gives rise to the question of whether therapeutic antibodies might interfere with the binding of diagnostic PD-L1 antibodies and might thereby potentially compromise monitoring of CTC PD-L1 expression in the course of therapy. 

In order to address this need for liquid biopsy—including CTC—standardization, several public–private partnerships and consortia were established: BloodPAC, European Liquid Biopsies Academy, Liquid Biopsy Consortium, SPIDIA4P, and the European IMI CANCER-ID consortium currently addressing—among others—the PD-L1 harmonization issue to detect PD-L1+ CTCs in NSCLC. In a collaboration project within the CANCER-ID consortium, we performed a comprehensive multicomparison of commercially available anti-PD-L1 antibodies in a NSCLC cell line panel including a preincubation with Atezolizumab (manuscript in preparation). Indeed, one of the major issues in translating PD-L1+ (circulating) tumor cells from basic research to the clinical setting for routine diagnostic application is (i) a heterogeneous detection rate of used CTC enrichment and detection approaches, (ii) resulting observer bias in calling CTCs, and (iii) the lack of consensus on the use of different commercially available anti-PD-L1 antibody clones and their performance and specificity compared to the antibody clones that are included in the IHC kits that received regulatory approval. 

### 3.1. The Need for Clinically Applicable CTC Enrichment and Detection Approaches

Today, there is no consensus on CTC enrichment in NSCLC patients resulting in the use of different enrichment strategies. The EpCAM-based CellSearch^®^ System which is FDA approved for clinical utility in metastasized breast, prostate, and colorectal cancer, remains challenging in NSCLC and studies using this system have to view with caution. To overcome the low sensitivity in advanced NSCLC patients using the CellSearch^®^ System, alternative methods for CTC detection were used in most studies (Table 1).

Recently, Janning and colleagues [29] compared the EpCAM-based CellSearch^®^ System with the epitope-independent Parsortix^TM^ system (Angle) for the assessment of PD-L1 expression of CTCs extracted from NSCLC patients. They showed a 50% higher detection rate of CTCs per blood sample with the Parsortix^TM^ system (>1 CTC in 59 of 97 samples (61%) compared to 31 of 97 samples (32%) with >1 CTC using the EpCAM-based system). Another promising method is the filter- and size-based ISET system which was used in several recent CTC-related studies in NSCLC patients (see Table 1). Similar to the Parsortix^TM^ system, an increased detection of CTCs compared to the CellSearch method was shown with the ISET method [39,40] as well as with a miniaturized microcavitiy array (MCA) [41]. 

Using the ISET method, Farace et al. [39] showed concordant results in only four patients (20%) while 16 (80%) patients had CTC counts markedly higher with ISET than CellSearch. In addition, Krebs and colleagues [40] detected 32 of 40 (80%) NSCLC patients using ISET compared with 9 of 40 (23%) patients using CellSearch. A subpopulation of CTCs isolated by ISET did not express epithelial markers. Using MCA, Hosokawa et al. [41] detected CTCs in 17 of 22 NSCLC patients using the MCA system versus 7 of 22 patients using the CellSearch system. On the other hand, CTCs were detected in 20 of 21 small cell lung cancer (SCLC) patients using the MCA system versus 12 of 21 patients with the CellSearch^®^ system. Significantly more CTCs in NSCLC patients were detected by the MCA system (median 13, range 0–291 cells/7.5 mL) than by the CellSearch^®^ system (median 0, range 0–37 cells/7.5 mL, *p* = 0.0015). However, statistical significance was not reached in SCLC, though the trend favoring the MCA system over the CellSearch^®^ system was observed (*p* = 0.2888). The MCA system also isolated CTC clusters from patients who had been identified as CTC negative using the CellSearch^®^ system [41]. 

Since most PD-L1 CTC studies use different enrichment techniques and methods for CTC detection, it becomes clear that comparisons between these studies (like those summarized in Table 1) have to be interpreted with caution. Additionally, the use of different anti-PD-L1 antibody clones reinforced this situation.

### 3.2. The Need for Harmonized Immunostaining Protocols

Numerous multicomparison studies already tried to clarify this situation with regards to IHC on tissue biopsy, summarized in Table 2. These reports concordantly highlight different immunostaining patterns, signal intensities, and therefore variable cut-off values regarding percentage of stained tumor or immune cells obtained by using the various antibody clones used.

Despite their scientific contribution on the topic, most PD-L1 analyses on CTCs were performed using different antibody clones (see Table 1)—with the only exception of Ilié and colleagues focusing on the clone SP142, which is part of the FDA approved Roche (Ventana) PD-L1 IHC assay. Furthermore, as Ilié et al. describe in their publication, some CTCs—as well as some screened tumor cell lines—exhibited cytoplasmic staining with or without a membranous signal, pointing out the necessity to extend the PD-L1 immunostaining assay to other clones.

## 4. Conclusions and Future Perspectives

Immune checkpoint inhibition therapy represents a breakthrough in treatment of non-small cell lung cancer patients. However, there are still major challenges in selecting NSCLC patients likely to benefit from targeting the PD-1/PD-L1 axis. CTC-based liquid biopsy may be an option for the development of blood-based tests that address this issue. The successful implementation of such tests will critically depend on consensus on the use of different anti-PD-L1 antibody clones, CTC enrichment technologies, and well-established standardized clinically feasible standard operating procedures. Furthermore, a deeper understanding of the different mechanisms of PD-L1 regulation at genetic, epigenetic, transcriptional, translational, and posttranslational levels in cancer is needed to develop appropriate protocols. In addition, the regulation of PD-L1 expression during metastasis might be different from the primary tumors, potentially making longitudinal monitoring of patients necessary and liquid biopsy an even more favorable diagnostic option. The latter is further supported when taking into account that PD-L1 expression assessed by IHC requires a tissue sample which could be insufficient, or even lacking, in advanced lung cancer patients. This may compromise the level of confidence with which a therapy decision can be made. Several studies suggest a promising role for PD-L1^+^ CTCs in determining response to different therapeutic approaches. However, the lack of consensus on anti-PD-L1 antibody clones persists, when most PD-L1 analyses on CTCs were performed with different antibody clones compared to tissue PD-L1 analysis. In addition, the value of CTC analysis for clinical practice is strongly determined by the sensitivity of the CTC isolation technology and the specificity of the diagnostic test to discriminate cells with malignant features from nonmalignant cells captured as background. To this end, the clinical benefit of immune checkpoint blockade in NSCLC using circulating tumor cells remains uncertain. CTCs have not been investigated in clinical trials relevant for regulatory approval of Atezolizumab, Nivolumab, and Pembrolizumab. More recently, a phase Ib study to evaluate safety and tolerability of durvalumab (anti-PD-L1) and tremelimumab (anti-CTLA-4) (NCT03275597) uses CTC number and CTC PD-L1 expression as exploratory endpoints for efficacy and target engagement. Future research will show whether CTC PD-L1 expression together with additional biomarkers like tumor mutational burden assessed by the analysis of CTCs or ctDNA, constitute clinically relevant blood-based biomarkers for immune checkpoint blockade therapy patient selection.

## Figures and Tables

**Table 1:** Studies on the clinical relevance of programmed death ligand 1-positive (PD-L1^+^) circulating tumor cells (CTCs) in non-small cell lung cancer (NSCLC)**.**

Study	Patients	Blood Tube	CTC-Enrichment System	Antibody Clone	Therapy	Clinical Outcome
Schehr et al. [23]	19	EDTA	Immunomagnetic depletion, Dynabeads-based	MIH1 (BD)	1st line TX: Radio-/ Chemotherapy, TKIs Current: ICIs	-
Bao et al. [24]	15	EDTA	Size-based (in-house produced chip)	*	1st line TX: Chemo Current: Nivolumab	-
Kallergi et al. [25]	30	EDTA	Size-based (ISET)	B7-H1 (NB)	1st line TX: None Current: Chemo-naïve	After 3 cycles of chemo, ~19% increase PD-L1^+^ CTCs
Adams et al. [26]	41	CellSave	Size-based (CellSieve Microfiltration Assay)	B7-H1 (R&D)	1st line TX: Chemo Current: Radiotherapy	Slightly better outcome in patients with high PD-L1 expression
Wang et al. [27]	13	EDTA	Microfluidic graphene oxide (GO) Chip	29E.2A3 (BL)	1st line TX: None Current: Radio-/ Chemotherapy	PD-L1^+^ patients had a shorter PFS compared to PD-L1^−^ patients
Ilié et al. [28]	106	-	Size-based (ISET)	SP142 (VT)	1st line TX: None Current: Chemo-naïve (93%), neoadjuvant chemo (7%)	Slightly better outcome in patients with PD-L1^+^ CTCs
Janning et al. [29]	89	EDTA and/or Cell Save	EpCAM-based (CellSearch^®^), size-based (Parsortix^TM^)	D84TX (CS)	Current: Radio-/ chemotherapy, surgery, TKIs, ICIs	Increase in PD-L1^+^ CTCs upon disease progression; no change or decrease in responding patients
Nicolazzo et al. [30]	24	CellSave	EpCAM-based (CellSearch)	B7-H1 (R&D)	1st line TX: na Current: Nivolumab	Poor clinical outcome
Guibert et al. [19]	96 pre-, 24 post- therapy	-	Size-based (ISET)	D8TX4 (CS)	1st line TX: Chemo Current: Nivolumab	More non-responders to Nivolumab if ≥1% PD-L1^+^ CTCs
Kulasinghe et al. [20]	33	EDTA or Streck	Size-based (ClearCell FX)	n/a (Abcam)	1st line TX: Radio-/ Chemotherapy Current: Nivolumab	None
Dhar et al. [33]	22	EDTA	Size-based (Vortex HT chip)	#4059 (PS),29E.2A3 (BL), MIH1 (BD)	1st line TX: na Current: ICIs	Slightly better outcome for patients with >50% PD-L1^+^ CTCs

Unless otherwise specified, CTC detection was performed via immunostaining; * CTC detection via RT-qPCR. Current therapy is defined as therapy at time point of blood draw. BD: BD Biosciences; BL: BioLegend; CS: Cell signaling; NB: Novus Biologicals; PS: ProSci; VT: Ventana. Chemo: Chemotherapy; na: Not available; TKI: Tyrosine kinase inhibitor; TX: Treatment.

**Table 2:** Harmonization studies on immunohistochemistry (IHC) PD-L1 staining of NSCLC tissue biopsies.

Study	Antibody Clone	Company	PD-L1 + Tumor Cell Cut-Off	Patients	Main Findings
Parra et al. [42]	E1L3N, E1J2J	Cell Signaling	≥1%	185 + (cell lines)	E1L3N, E1J2J, SP142, 28-8, 22C3, 5H11 and SP263: comparable staining patterns on membranes; SP263: higher IHC score
	22C3, 28-8	Dako			
	SP263, SP142	Ventana			
	5H11	Not commercialized			
Ratcliffe et al. [43]	22C3, 28-8	Dako	≥1%, ≥10%, ≥25%, ≥50%	493	All assays show concordant staining patterns
	SP263	Ventana			
Scheel et al. [44]	E1L3N	Cell Signaling	≥1%, ≥50%	21	22C3, 28-8 and SP263: concordant staining patterns; SP142 as outlier
	22C3, 28-8	Dako			
	SP263, SP142	Ventana			
Adam et al. [45]	E1L3N	Cell Signaling	≥1%, ≥5%, ≥25%, ≥50%	41	28-8, 22C3, SP263, E1L3N: highly concordant; SP142 as outlier
	22C3, 28-8	Dako			
	SP263, SP142	Ventana			
Rimm et al. [46]	E1L3N	Cell Signaling	≥1%, ≥5%, ≥50%	90	SP142: significant lower PD-L1 IHC score; 22C3: significant reduction in PD-L1 staining; 28-8 and E1L3N concordant
	22C3, 28-8	Dako			
	SP142	Ventana			

## Supplementary Materials

The following are available online, Table S1: Clinical trials in lung cancer (drug: Nivolumab), Table S2: Clinical trials in lung cancer (drug: Atezolizumab), Table S3: Clinical trials in lung cancer (drug: Pembrolizumab).
