# Supplementary material for: Circulating Tumor Cell PD-L1 Expression as Biomarker for Therapeutic Efficacy of Immune Checkpoint Inhibition in NSCLC
Source: Cells. 2019 Aug 1;8(8):809. doi: 10.3390/cells8080809 (PMC6721635; doi:10.3390/cells8080809)
Supplement: Supplementary file 1 [file cells-08-00809-s001.pdf]

Table S1: Clinical trials in lung cancer (drug: Nivolumab)

Terms	Search Results*	Entire Database**
Synonyms		
**Nivolumab**	203 studies	959 studies
Opdivo	107 studies	532 studies
MDX 1106	34 studies	179 studies
**Lung Cancer**	203 studies	6,881 studies
Lung Neoplasm	184 studies	5,922 studies
Lung carcinoma	46 studies	1,038 studies
CARCINOMA OF LUNG	3 studies	19 studies
Neoplasm of lung	2 studies	27 studies
Cancer of the Lung	1 studies	36 studies
Cancer of Lung	--	17 studies
cancers lungs	--	4 studies
Carcinoma of the Lung	--	43 studies
lung malignancies	--	5 studies
lung malignant tumors	--	1 studies
lung tumors	--	75 studies
Neoplasm of the lung	--	1 studies
pulmonary cancer	--	20 studies
Pulmonary neoplasia	--	1 studies
Pulmonary Neoplasm	--	27 studies
Tumor of lung	--	1 studies
Tumor of the Lung	--	2 studies
**Cancer**	203 studies	68,344 studies
Neoplasm	199 studies	59,774 studies
Tumor	49 studies	15,243 studies
Malignancy	11 studies	2,918 studies
Oncology	7 studies	1,082 studies
neoplastic syndrome	1 studies	576 studies
Neoplasia	--	584 studies
Neoplastic Disease	--	19 studies
**Lung**	203 studies	21,049 studies
Pulmo	--	2 studies
pulmonary	--	7,451 studies

All types of lung cancer were included; -- No studies found; *Number of studies in the search results containing the term or synonym; **Number of studies in the entire database containing the term or synonym

Table S2: Clinical trials in lung cancer (drug: Atezolizumab)

Terms	Search Results*	Entire Database**
Synonyms		
**Atezolizumab**	97 studies	348 studies
Tecentriq	43 studies	157 studies
MPDL3280A	39 studies	123 studies
anti-PDL1	15 studies	55 studies
RG7446	7 studies	36 studies
MPDL 328OA	5 studies	33 studies
**Lung Cancer**	97 studies	6,881 studies
Lung Neoplasm	94 studies	5,922 studies
Lung carcinoma	13 studies	1,038 studies
CARCINOMA OF LUNG	2 studies	19 studies
Cancer of the Lung	1 studies	36 studies
lung tumors	1 studies	75 studies
Cancer of Lung	--	17 studies
cancers lungs	--	4 studies
Carcinoma of the Lung	--	43 studies
lung malignancies	--	5 studies
lung malignant tumors	--	1 studies
Neoplasm of lung	--	27 studies
Neoplasm of the lung	--	1 studies
pulmonary cancer	--	20 studies
Pulmonary neoplasia	--	1 studies
Pulmonary Neoplasm	--	27 studies
Tumor of lung	--	1 studies
Tumor of the Lung	--	2 studies
**Cancer**	97 studies	68,344 studies
Neoplasm	96 studies	59,774 studies
Tumor	15 studies	15,243 studies
Malignancy	1 studies	2,918 studies
neoplastic syndrome	1 studies	576 studies
Oncology	1 studies	1,082 studies
Neoplasia	--	584 studies
Neoplastic Disease	--	19 studies
**Lung**	97 studies	21,049 studies
Pulmo	--	2 studies
pulmonary	--	7,451 studies

All types of lung cancer were included; -- No studies found; *Number of studies in the search results containing the term or synonym; **Number of studies in the entire database containing the term or synonym

Table S3: Clinical trials in lung cancer (drug: Pembrolizumab)

Terms	Search Results*	Entire Database**
Synonyms		
**Pembrolizumab**	225 studies	1,024 studies
Keytruda	115 studies	582 studies
MK-3475	111 studies	513 studies
lambrolizumab	27 studies	153 studies
**Lung Cancer**	225 studies	6,881 studies
Lung Neoplasm	202 studies	5,922 studies
Lung carcinoma	47 studies	1,038 studies
Carcinoma of the Lung	2 studies	43 studies
Neoplasm of lung	2 studies	27 studies
Cancer of the Lung	1 studies	36 studies
CARCINOMA OF LUNG	1 studies	19 studies
Cancer of Lung	--	17 studies
cancers lungs	--	4 studies
lung malignancies	--	5 studies
lung malignant tumors	--	1 studies
lung tumors	--	75 studies
Neoplasm of the lung	--	1 studies
pulmonary cancer	--	20 studies
Pulmonary neoplasia	--	1 studies
Pulmonary Neoplasm	--	27 studies
Tumor of lung	--	1 studies
Tumor of the Lung	--	2 studies
**Cancer**	225 studies	68,344 studies
Neoplasm	216 studies	59,774 studies
Tumor	79 studies	15,243 studies
Malignancy	10 studies	2,918 studies
Oncology	8 studies	1,082 studies
neoplastic syndrome	1 studies	576 studies
Neoplasia	--	584 studies
Neoplastic Disease	--	19 studies
**Lung**	225 studies	21,049 studies
pulmonary	1 studies	7,451 studies
Pulmo	--	2 studies

All types of lung cancer were included; -- No studies found; *Number of studies in the search results containing the term or synonym; **Number of studies in the entire database containing the term or synonym
